# Analysis of the use of educational materials by radiologists and radiology residents in Brazil: paradigm shift

**DOI:** 10.1590/0100-3984.2021.0107

**Published:** 2022

**Authors:** Letícia Motono Chojniak, Rony Klaus Isberner, Juliana de Oliveira Souza, Valdair Francisco Muglia, Almir Galvão Vieira Bitencourt, Rubens Chojniak

**Affiliations:** 1 Faculdade de Medicina da Universidade Nove de Julho (Uninove), São Paulo, SP, Brasil.; 2 Universidade do Vale do Itajaí (Univali), Itajaí, SC, Brasil.; 3 A.C.Camargo Cancer Center, Departamento de Imagem, São Paulo, SP, Brasil.; 4 Faculdade de Medicina de Ribeirão Preto da Universidade de São Paulo (FMRP-USP), Ribeirão Preto, SP, Brasil.

**Keywords:** Radiology, Teaching materials, Education, continuing, Multimedia, Radiologia, Materiais de ensino, Educação continuada, Multimídia

## Abstract

**Objective:**

To profile the use of educational materials by radiologists and radiology residents in Brazil.

**Materials and Methods:**

This was a cross-sectional, descriptive, observational study in which an electronic questionnaire was sent via email to physicians working in the field of radiology. The questionnaire contained questions regarding the profile, education, and place of work of the physicians, as well as their access to educational resources and the types of educational materials they used most.

**Results:**

The questionnaire was completed by 265 physicians. The mean age of the respondents was 38.2 years. Of the 265 respondents, 212 (80.0%) worked with ultrasound, 206 (77.7%) worked with computed tomography, 170 (64.2%) were board-certified, 166 (62.6%) were male, 174 (65.7%) worked at private facilities, and 167 (63.0%) had no academic affiliation. The mean weekly total study time was 9.6 h (median, 6 h), being higher among physicians in training, among those who worked at public facilities, and among those who worked at teaching hospitals. Regarding the device employed in order to access educational materials, there was a trend toward greater use of computers, which were employed by 65.3%. The majority of the respondents (61.9%) chose to access educational materials that were free of charge, the most common sources being websites, eBooks, and online journals.

**Conclusion:**

Electronic and digital resources are the main means of access to educational materials used by radiologists in Brazil, the most commonly used resources being web sites, eBooks, and online journals.

## Abstract (pt)

**Objetivo:**

Identificar o perfil de utilização dos recursos didáticos pelos médicos radiologistas e residentes em radiologia e diagnóstico por imagem brasileiros.

**Materiais e Métodos:**

Estudo observacional, transversal e descritivo, realizado por meio de questionário eletrônico contendo perguntas referentes ao perfil do médico, formação, local de atuação, perfil de acesso a recursos educacionais e tipos de recursos mais utilizados.

**Resultados:**

Responderam ao questionário 265 médicos com idade média de 38,2 anos, sendo a maioria radiologistas formados (170; 64,2%), do sexo masculino (166; 62,6%), trabalhando em instituições privadas (174; 65,7%), sem vínculo acadêmico (167; 63,0%), atuando principalmente nas áreas de ultrassonografia (212; 80,0%) e tomografia computadorizada (206; 77,7%). A carga horária média de estudo semanal foi de 9,6 horas (mediana: 6 horas), sendo maior no grupo dos médicos que estão em formação e nos que atuam em instituições públicas e acadêmicas. Quanto ao recurso para o acesso do material de estudo, observou-se uma tendência pela maior utilização do computador (65,3%). A maioria dos respondentes optou por acessar materiais didáticos sem custo (61,9%), sendo os *sites* da *web*, livros e revistas eletrônicas os mais pesquisados.

**Conclusão:**

Os radiologistas brasileiros utilizam recursos eletrônicos e digitais como principais materiais didáticos, sendo os *sites* da *web*, livros e revistas eletrônicas as fontes mais utilizadas.

## INTRODUCTION

The technological evolution that occurred in various medical fields over the last 35 years was mainly driven by digital automation, which has resulted in structural changes in the entire service network, as well as in the behavior of health care professionals, who needed to adapt to the innovations of the digital age. Diagnostic imaging was one of the medical specialties most affected by that evolution, because of the introduction of new digital equipment and the need for professionals in order to improve the technological resources, and a culture of continuing education developed. Digital literacy became fundamental for that process, not only in medical schools, but also for health care professionals already in the job market.

New technologies and their innovations have revolutionized the way people communicate, thus increasing the quantity and quality of the information available, as well as the speed of information exchange^(1)^. Prior to the widespread adoption of electronic media, most educational resources in the field of radiology were print books and print journals. The need for broader, faster searches for information in the area of diagnostic imaging prompted radiologists and radiology residents to adopt the use of other media resources, such as computers, cell phones, and tablets^(2)^.

Virtual tools and environments have been shown to play an important role in learning^(3)^. Mobile technologies are now increasingly used by students and educators around the world, in order to access information, rationalize/simplify time management, and facilitate learning in an innovative way^(4,5)^. The use of web platforms to interact, generate, access, and disseminate information has become common practice among health care professionals^(6-9)^.

When it is necessary to obtain information quickly and easily, the “technological environment” modifies the behavior of health care professionals in their daily lives, putting them into a very close relationship with the available digital resources. That interaction breaks paradigms and aids in the personal and professional development of the individual. The observation of radiology residents and radiologists leads us to suspect that the educational reference materials used have shifted from standard print material to internet resources. However, there have been few studies documenting that change in behavior.

Knowledge of the preference for and frequency of use of the various sources of print information or electronic media consulted by radiologists who work in various settings and types of institutions, at different stages in their careers, could be useful for specialty societies, coordinators of training programs, and managers of major radiology centers. Such knowledge would make it possible to outline a strategy for providing access to educational resources, in terms of the choice of books, internet resources, and licenses for selected sites, as well as providing specialist physicians and residents in the area with information regarding the use of resources and the preferences of their colleagues. Therefore, the objective of this study was to profile the use of educational resources by radiologists and radiology residents in Brazil.

## MATERIALS AND METHODS

This was a cross-sectional, predominantly quantitative, observational study, with descriptive characteristics. Prospective respondents were sent, via e-mail, an invitation letter, an informed consent form, and an electronic questionnaire on the use of educational resources, through the communication channel of the Teaching, Improvement, and Medical Residency Committee of the Colégio Brasileiro de Radiologia e Diagnóstico por Imagem (CBR, Brazilian College of Radiology and Diagnostic Imaging), which brings together radiologists (members of the CBR) and resident physicians at radiology and diagnostic imaging centers affiliated with the CBR.

The questionnaire was sent via the Google forms tool, for data distribution and collection, and comprised the following items:

1. Work profile:
  - Age;
  - Sex (female;male);
  - Training/specialization;
  - Length of experience in diagnostic imaging;
  - Main modalities/areas of activity;
  - Academic degree (none, master’s, doctoral, or tenured professorship);
  - State of practice;
  - Profile of main workplace (clinic vs. hospital, public vs. private, and teaching vs. non-teaching).
2. Profile of access to educational materials:
  - Estimated total time (in hours) spent studying/consulting educational material per week;
  - Access to educational resources, including print books, eBooks, print journals, online journals, websites, social networks, and mobile applications;
  - The device most commonly used in order to access digital educational materials (computer, tablet, cell phone, or none);
  - The most commonly accessed print textbooks or journals/websites/applications;
  - Institutional access to any paid material/website/educational program at the main place of work.

On the questions related to a preference for certain educational resources, respondents were allowed to provide multiple answers, as well as to write in answers not included in the list of options.

A total of 265 professionals, including radiology residents and board-certified radiologists, some with specialist titles or a certificate of competence in diagnostic imaging, completed and returned the questionnaire. All of the respondents gave written informed consent for the anonymous use of their data. On the basis of the data obtained, descriptive statistics parameters were used, the usual measures of central tendency being adopted, together with the calculation of absolute and relative frequencies. Groups and subgroups were created according to the following selected categories: level of training—physicians in training (first-, second-, third-, or fourth-year residents) and trained physicians; academic degree—master’s, doctoral, or tenured professorship); type of workplace (public or private facility); frequency of use of resources (daily, weekly, monthly/rarely, or never); number of physicians working at the facility; regional human development index (HDI)—high HDI (southern, southeastern, or central-west region) or low HDI (northern or northeastern region); generation—Generation Y (< 40 years of age) or Generation X/Baby Boomers (≥ 40 years of age); simple frequency crossings between the level of training and the types of media used (journals, books, websites, social networks, and applications); absolute and relative frequency of region by HDI; frequency of each type of device used in order to access digital material (computer, tablet, and cell phone); and the specific source of each journal, website, and application.

The analysis to determine the relationship among the subgroups for categorical variables was performed with Pearson’s chi-square test and Fisher’s exact test. For continuous variables, the Kruskal-Wallis test and Mann-Whitney U test were used. The information obtained from the questionnaires was tabulated in electronic spreadsheets and subsequently analyzed for significance in the various intersections. Values of *p* < 0.05 were considered significant. Questions that were unanswered were not included in the analysis.

## RESULTS

Table 1 shows the characteristics of the 265 physicians who completed the questionnaire. The mean age of the respondents was 38.2 years (median, 35 years), and the mean length of experience was 10.8 years (median, 7 years). Of the 265 respondents, 170 (64.2%) were trained physicians, including 136 (51.3%) with specialist titles, and 95 (35.8%) were physicians in training, first-, second-, third-, and fourth-year residents accounting for 20, 22, 28, and 25, respectively. The area of expertise most often cited was ultrasound (in 80.0%), followed by computed tomography (in 77.7%), magnetic resonance imaging (in 59.2%), radiography (in 49.4%), mammography (in 32.1%), densitometry (in 17.7%), interventional radiology (in 10.9%), and nuclear medicine (in 1.5%). The region of Brazil that accounted for the greatest proportion of respondents was the southeastern region, where, 155 (58.5%) of the respondents worked, whereas 40 (15.1%) worked in the southern region, 39 (14.7%) worked in the northeastern region, 22 (8.3%) worked in the central-west region, and only nine (3.4%) worked in the northern region.

**Table 1:** Profile of the physicians participating in the study (N = 265).

Variable	Categories	%	n
Sex	Male	62.6	166
	Female	37.4	99
Status	In training	35.8	95
	Training completed	64.2	170
Age (years)	≤ 40	69.4	184
	> 40	30.6	81
Graduate degree	No	83.4	221
	Master’s	7.9	21
	Doctoral	7.5	20
	Tenured professorship	1.1	3
Regional HDI	High	81.9	217
	Low	18.1	48
Profile of the main workplace	Clinic	36.6	97
of the respondent	Hospital	63.4	168
	Public	34.3	91
	Private	65.7	174
	Teaching	37.0	98
	Non-teaching	63.0	167
Device employed*	Computer	65.3	173
	Cell phone	22.3	59
	Tablet	12.1	32
Use of paid material^†^	Yes	33.2	88
	No	61.9	164

* One respondent did not answer.

† Thirteen respondents did not answer.

The educational resources most often used were websites (used by 92.4%), eBooks (used by 66.4%), online journals (used by 61.1%), applications (used by 60.7%), social networks (used by 57.3%), print books (used by 47.9%), and print journals (used by 16.9%). Table 2 presents information about the journals, websites, and applications most often accessed by the respondents. Table 3 shows the correlations that the variables educational status (radiology resident or radiologist), age, and regional HDI had with the variables, academic degree, profile of the main workplace, digital access, use of paid material, and educational resources.

**Table 2:** Journals, websites, and applications most often accessed by the respondents (N = 265).

Category	Name	%	n
Journals	*Radiographics*	77.4	205
	*Radiology*	71.7	190
	*Radiologia Brasileira*	63.0	167
	*American Journal of Roentgenology*	50.9	135
	*Journal of the American College of Radiology*	38.1	101
	*European Radiology*	35.1	93
	*European Journal of Radiology*	34.3	91
Websites/	Radiopaedia	78.9	209
networks	CBR	67.9	180
	Google Scholar	60.4	160
	Sociedade Paulista de Radiologia	60.0	159
	IMAIOS	59.6	158
	Workplace	57.7	153
	PubMed	53.2	141
	STATdx	39.2	104
	American College of Radiology	39.2	104
	Facebook	36.2	96
	Learning Radiology	33.2	88
	AuntMinnie	32.5	86
	UpToDate	25.3	67
	eMedicine	15.5	41
Applications	e-Anatomy	43.8	116
	Radiology Assistant 2.0	31.3	83
	CTisus	11.7	31
	Diagnostic Radiology	11.3	30
	Thoracic Imaging	9.8	6
	MR Imaging in Prostate Cancer	7.5	20

**Table 3:** The variables radiology training status, age, and regional HDI, correlated with degree, profile of the main workplace of the respondent, device employed, use of paid material, and educational resources accessed by the respondents (N = 265).

Variable	Categories	Training status				*P*	Age (years)				*P*	HDI				*P*
		In training		Completed			≤ 40		> 40			High		Low		
		%	n	%	n		%	n	%	n		%	n	%	n	
Graduate degree	No	97.9	93	75.3	128		92.4	170	63.0	51		82.0	178	89.6	43	
	Yes	0.2	2	24.7	42	*	7.6	14	37.0	30	*	18.0	39	10.4	5	ns
Profile of the main work-	Clinic	13.7	13	49.4	84		29.3	54	53.1	43		35.5	77	41.7	20	
place of the respondent	Hospital	86.3	82	50.6	86	*	70.7	130	46.9	38	*	64.5	140	58.3	28	ns
	Public	52.6	50	24.1	41		38.6	71	24.7	20		32.3	70	43.8	21	
	Private	47.4	45	75.9	129	*	61.4	113	75.3	61	*	67.7	147	56.3	27	ns
	Teaching	58.9	56	24.7	42		42.4	78	24.7	20		38.2	83	31.3	15	
	Non-teaching	41.1	39	75.3	128	*	57.6	106	75.3	61	*	61.8	134	68.8	33	ns
Device employed	Computer	62.8	59	67.1	114		63.9	117	69.1	56		70.4	152	43.8	21	
	Cell phone	19.1	18	24.1	41		22.4	41	22.2	18		20.8	45	29.2	14	
	Tablet	18.1	17	8.8	15	ns	13.7	25	8.6	7	ns	8.8	19	27.1	13	*
Use of paid material	Yes	42.0	37	31.1	51		38.7	67	26.6	21		37.1	76	25.5	12	
	No	58.0	51	68.9	113	ns	61.3	106	73.4	58	ns	62.9	129	74.5	35	ns
Educational resources	Print books	65.3	62	57.6	98		59.2	109	63.0	51		59.5	130	62.5	30	
	eBooks	90.5	86	73.5	125	ns	58.8	156	67.9	55	ns	78.8	171	83.3	40	ns
	Print journals	34.7	33	38.8	66		32.6	60	48.2	39		38.7	84	31.3	15	
	Online journals	28.3	5	53.6	142	ns	54.0	143	27.9	74	ns	66.8	177	15.1	40	ns
	Websites	97.9	93	94.7	161		97.8	180	91.4	74		94.9	206	100.0	48	
	Social networks	78.9	75	58.8	100		71.2	131	54.3	44		67.7	147	58.3	28	
	Applications	78.9	75	63.5	108	ns	75.0	138	55.6	45	ns	66.8	145	79.2	38	ns

* Significant—chi-square test (*p* < 0.05). ns, not significant.

As can be seen in Table 4, the mean time spent studying per week was 9.6 hours (median, 6 hours), although the time was longer among the physicians in training and among those whose main workplace was a hospital (rather than a clinic), a public facility (rather than a private facility), or a teaching hospital (rather than a non-teaching hospital). Physicians who had completed their residency or who worked in regions of the country with a high HDI reported using a computer more often than they used a cell phone or tablet (Table 5).

**Table 4:** Number of hours per week spent studying educational materials, by sex, training status, age, graduate degree, regional HDI, and profile of the main workplace of the respondents.

Variable	Categories	Hours of study per week		*P*
		Mean	Median	
Sex	Male	8.7	6	ns
	Female	11.2	5	
Status	In training	14.3	10	*
	Training completed	7.1	5	
Age (years)	≤ 40	10.2	7	ns
	> 40	8.2	5	
Graduate degree	No	10.1	6	ns
	Master’s	5.1	5	
	Doctoral	8.7	7	
	Tenured professorship	7.5	7.5	
Regional HDI	High	9.9	6	ns
	Low	8.3	6	
Profile of the main workplace of the respondent	Clinic	5.8	4	*
	Hospital	11.7	8	
	Public	12.8	8	*
	Private	7.9	5	
	Teaching	13.7	8	*
	Non-teaching	7.1	5	

* Significant—chi-square test (*p* < 0.05). ns, not significant.

**Table 5:** Profile of the respondents (N = 265), stratified by the device employed for digital access.

Variable	Categories	Way of digital access						*P*
		Computer		Cell phone		Tablet		
		%	n	%	n	%	n	
Sex	Male	40.8	108	15.1	40	6.8	18	ns
	Female	24.5	65	7.2	19	5.7	15	
Status	In training	22.3	59	6.8	18	6.8	18	*
	Training completed	43.0	114	15.5	41	5.7	15	
Age (years)	≤ 40	44.2	117	15.5	41	9.8	26	ns
	> 40	21.1	56	6.8	18	2.6	7	
Graduate degree	No	51.3	136	20.0	53	12.1	32	†
	Master’s	6.8	18	1.1	3	0.0	0	
	Doctoral	6.4	17	1.1	3	0.0	0	
	Tenured professorship	0.8	2	0.0	0	0.4	1	
Regional HDI	High	57.4	152	17.0	45	7.5	20	*
	Low	7.9	21	5.3	14	4.9	13	
Profile of the main workplace of the respondent	Clinic	23.4	62	9.8	26	3.4	9	ns
	Hospital	41.9	111	12.5	33	9.1	24	
	Public	20.8	55	7.9	21	5.7	15	ns
	Private	44.5	118	14.3	38	6.8	18	
	Teaching	24.9	66	7.2	19	4.9	13	ns
	Non-teaching	40.4	107	15.1	40	7.5	20	
Use of paid material	Yes	24.5	65	4.9	13	3.8	10	ns
	No	37.4	99	17.0	45	7.5	20	
Educational resources	Print books	40.8	108	12.8	34	6.8	18	ns
	eBooks	50.6	134	18.5	49	10.6	28	ns
	Print journals	26.0	69	8.3	22	3.0	8	ns
	Online journals	57.4	152	15.8	42	8.7	23	*
	Websites	62.6	166	21.9	58	11.3	30	ns
	Social networks	40.0	106	17.4	46	8.7	23	*
	Applications	41.1	109	18.9	50	9.1	24	*

* Significant—chi-square test (*p* < 0.05). ns, not significant.

† None vs. master’s, doctoral, or tenured professorship.

## DISCUSSION

There are few data in the existing literature regarding the profile of radiologists, in Brazil or elsewhere, particularly in relation to the types of educational resources they use. This study profiles the use of educational resources by physicians in training and physicians trained in the field of diagnostic imaging, as well as the resources and types of media most often used by such physicians, together with some characteristics of the activities carried out by these professionals in their daily routine. Most of the respondents had completed their residency, were male, were under 40 years of age, had no graduate degree, worked in a region of Brazil with a high HDI, worked at a private hospital or clinic, had no academic affiliation, and had expertise mainly in the field of ultrasound or computed tomography. As a means of accessing the educational material, there was a trend towards greater use of computers (rather than cell phones or tablets). Most of the respondents choose to access educational materials that were available at no cost, websites and online journals being the sources most often accessed.

The demographic characteristics of the respondents in our sample were in line with those reported in a recent survey conducted by the CBR, involving 12,868 radiologists (62.4% men), among whom the mean age was 46.1 years and 54.0% were under 40 years of age^(10)^. In that study, as in ours, there was a greater number of physicians working in regions of the country with a high HDI, mainly in the southeastern region, especially among the radiologists in training, which may be related to the greater number of medical residency vacancies in diagnostic imaging in such regions. In addition, the technologically complex aspects of radiology are taught and practiced primarily in large cities, at specialized, regionalized, or referral facilities^(10)^.

In our sample, the physicians interviewed studied an average of 9.6 h per week. For those who worked at public hospitals and those with an academic affiliation, the mean weekly study time was nearly double that reported by those who worked at private clinics or hospitals. Overall, the mean weekly study time was longest among the residents.

In our sample, the type of device used in order to access digital material was a computer, followed by a cell phone and a tablet. Some authors have conducted comparative studies of the use of mobile and fixed devices, in which factors such as socioeconomic status, skill level, and limited access to web resources via mobile devices influenced the decision to use a computer, as did the need to have a screen with a wider field of view^(11)^. It is noteworthy that most health care professionals working in the field of diagnostic imaging already use computers to view images on larger screens and can use that same device for their studies. We found that the tendency to use cell phones and tablets was greater among the health care professionals who were under 40 years of age, the use of tablets being greatest among the youngest physicians and residents. A survey conducted by the Internet Steering Committee in Brazil, designated TIC Kids Online Brazil 2017, indicated that computers (desktops, laptops, and tablets) are losing ground, showing that the proportion of children using computers to access the Internet dropped from 90% in 2013 to 53% in 2017^(12)^. These data demonstrate a trend toward a preference for the use of mobile devices (especially cell phones) by the younger generations.

The ease of access and searching with digital tools, as well as the possibility of reading on different devices such as cell phones, tablets, and computers, make these resources highly attractive^(13-15)^. However, some factors can be considered barriers to online research, such as the need to use passwords or usernames, too much information to digitize and little specific information to answer a defined question, the credibility of the source accessed, and having to pay for content^(16)^.

Most of our respondents stated that they prefer to use open access materials. However, the younger respondents (physicians in training) were slightly more likely to access paid content than were the radiologists who were over 40 years of age. There is a global trend toward making educational tools available at no cost, such tools already being standardized, collectively having been given the generic name of free open access medical (FOAM) education^(17)^. Websites were the sources most often utilized in searches, being accessed by 254 of the 265 respondents. Although there was broad variation in the types of websites accessed, the predominant ones were those of specialty societies, scientific journals, and educational institutions; the point of convergence was the search for websites of highly credible institutions. There was also an impressive number of views of online journals and eBooks, followed by applications and social networks, mainly by the younger respondents. There was a trend toward greater use of applications and social networks by younger (Generation Y) respondents, those who were physicians in training, those who worked at public facilities, and those who worked in a region with a high HDI, the daily frequency of access, notably to social networks, in which residents were more significantly engaged, being higher among such respondents.

Social networks can be quite useful as research tools because of their great popularity and widespread use, in Brazil and elsewhere, having a wide reach due to the number of connections established among the people who use them. In addition, the use of a social network is an easy-to-use, low-cost method of rapidly disseminating information^(18,19)^. It is noteworthy that the global use of mobile devices, with their capacity for connectivity, integrated with the possibilities of social media, also provides a rich platform for innovative scientific experiences in student-directed learning^(20)^.

In our survey, scientific journals in the field of diagnostic imaging were also cited as common research sources. Among such journals, those most frequently accessed were *Radiographics, Radiology, Radiologia Brasileira*, and the *American Journal of Roentgenology*. Most of those journals publish review articles, which are generally the article type most sought after by health care professionals in training or seeking retraining. It is noteworthy that the journal most accessed by the respondents—*Radiographics*—publishes exclusively review articles and pictorial essays. In our sample, those research sources were typically accessed by radiologists who were under 40 years of age, worked in a region with a high HDI (especially in cities with more than a million inhabitants), and worked at a facility with more than 20 radiologists on staff, a computer being the device most often used to access them.

Our study has some limitations. The data sample, obtained from answers to questionnaires sent via an online platform, was small in relation to the total number of radiologists working in Brazil. In addition, the number of respondents in certain subgroups was small. Furthermore, it is possible that the radiologists who completed the questionnaire were those with the greatest interest in continuing education and digital education, which could represent a self-selection bias. For some variables, certain approximations and generalizations were allowed. For example, the geographic regions of the country were stratified by HDI, although it is known that there are areas of high HDI in regions with low development and vice versa.

In conclusion, our findings indicate that, on average, radiologists currently spend fewer than 10 h per week engaged in the study of educational material, although that number appears to be higher among radiology residents, as well as among those who work at hospitals (especially teaching hospitals) and public facilities. Our data also suggest that radiologists, while still in training or after graduation, predominantly use electronic and digital resources to access sources of educational material. In the present study, we have demonstrated the importance of using technological resources to obtain information in the field of diagnostic imaging by showing that the vast majority of our respondents employed such resources. Websites and online journals appear to be the most researched type of media most often utilized by radiologists and radiology residents. In addition, there appears to be a tendency for such professionals to use a computer to access digital media and to choose open access content over paid content.
